# An interdisciplinary review of digital technologies to facilitate anti-corruption, transparency and accountability in medicines procurement

**DOI:** 10.1080/16549716.2019.1695241

**Published:** 2020-03-20

**Authors:** Tim K. Mackey, Raphael E. Cuomo

**Affiliations:** a Global Health Policy Institute, San Diego, CA, USA; b Department of Anesthesiology and Division of Infectious Diseases and Global Public Health, University of California, San Diego – School of Medicine, San Diego, CA, USA; c Department of Healthcare Research and Policy, University of California, San Diego – Extension, San Diego, CA, USA

**Keywords:** Anti-Corruption, Transparency and Accountability, Medicines procurement, corruption, technology, e-procurement, transparency, access to medicines, global health

## Abstract

**Background**: Pharmaceutical corruption is a serious challenge in global health. Digital technologies that can detect and prevent fraud and corruption are particularly important to address barriers to access to medicines, such as medicines availability and affordability, stockouts, shortages, diversion, and infiltration of substandard and falsified medicines.

**Objectives**: To better understand how digital technologies are used to combat corruption, increase transparency, and detect fraud in pharmaceutical procurement systems to improve population health outcomes.

**Methods**: We conducted a multidisciplinary review of the health/medicine, engineering, and computer science literature. Our search queries included keywords associated with medicines procurement and digital technology in combination with terms associated with transparency and anti-corruption initiatives. Our definition of ‘digital technology’ focused on Internet-based communications, including online portals and management systems, supply chain tools, and electronic databases.

**Results**: We extracted 37 articles for in-depth review based on our inclusion criteria focused on the utilization of digital technology to improve medicines procurement. The vast majority of articles focused on electronic data transfer and/or e-procurement systems with fewer articles discussing emerging technologies such as machine learning and blockchain distributed ledger solutions. In the context of e-procurement, slow adoption, justifying cost-savings, and need for technical standards setting were identified as key challenges for current and future utilization.

**Conclusions**: Though there is a significant promise for digital technologies, particularly e-procurement, overall adoption of solutions that can enhance transparency, accountability and concomitantly combat corruption, is still underdeveloped. Future efforts should focus on tying cost-saving measurements with anti-corruption indicators, prioritizing centralization of e-procurement systems, establishing regulatory harmonization with standards setting, and incorporating additional anti-corruption technologies into procurement processes for improving access to medicines and to reach the overall goal of Universal Health Coverage.

## Background

Each year an estimated $7.5 trillion is spent worldwide on providing health services, yet as much as 6% or $300 billion is lost to corruption and errors [1,2]. In developed countries, the cost of corruption is estimated to be from $12 to $23 billion per year [1]. Corruption, the misappropriation of authority, resources, trust or power for private or institutional gain, is particularly problematic for public procurement, where it is estimated that 10% to 30% of public procurement spending is lost to mismanagement and corruption [3,4]. This risk also extends to the complex and global ecosystem of medicines procurement, where the consequences of corruption in the drug supply chain can directly impact patient and population health by limiting equitable, safe, and affordable access to medicines.

Access to medicines is generally defined by the World Health Organization (WHO) as ‘having medicines continuously available and affordable at public or private health facilities …’ (which also includes access to all health products and medical devices) and is underpinned by a framework of promoting rational selection, affordable prices, sustainable financing, and reliable health and supply systems [5–7]. Importantly, improving access to medicines through anti-corruption initiatives is now on the global health agenda with international stakeholders convening to establish a Global Network on Anti-Corruption, Transparency and Accountability in February 2019. These anti-corruption activities, also known by the term ‘ACTA’, are critical as corruption in pharmaceutical procurement can undermine all four elements of the access to medicines framework [8].

Specifically, the large amounts of public spending and necessity to procure services and products for complex health decision-making and service delivery give rise to opportunities for corruption to infiltrate local, state, regional, and international healthcare sectors. Within the healthcare sector, the pharmaceutical sector meets all the enabling criteria of a highly regulated and complex industry, which also includes the participation of multiple public and private stakeholders (e.g. financers, suppliers, manufacturers, wholesalers, logistic providers, distributors, and prescribers/dispensers) who have their own incentives and rules across the supply chain. All of these stakeholders have high levels of discretion at multiple decision points that can be susceptible to corruption in the absence of adequate transparency and accountability [3].

The overall goal of medicines procurement is to ensure that a product is purchased in the right quantity and quality at a price that is cost effective and is available at the time required, objectives that are again directly undermined by the presence of corruption [9]. Specific forms of corruption that occur in medicines procurement processes include process rigging, collusion, and fraud between and among public and private actors. These corrupt acts can result in unfair competition in the tendering process, bribery, cartelism, kickbacks, and overpayments and false invoicing [10]. Corrupt practices may manifest themselves differently depending on the phase of procurement (e.g. pre-bidding stage, bidding stage, post-bidding/award stage, and implementation stage), with each phase representing its own unique set of vulnerabilities [11].

The consequences of corruption, driven largely by lack of transparency in the medicines procurement cycle, can also include loss of trust in public institutions by citizens, increased budgetary constraints from unnecessary spending and higher priced medicines, suboptimal procurement decision-making, delays in medicines procurement, drug diversion, exposure to substandard and falsified medicines, and other market failures [12]. The ultimate consequence of these breakdowns in drug procurement can lead to negative community impact, such as failure to provide needed treatment, higher out-of-pocket expenditures, reduction in health-seeking behavior, adverse health events, and even patient death [13–17].

## Objective

To address concerns about corruption and fraud in pharmaceutical procurement, advances in information systems, telecommunication networks, web and cloud-based platforms, and big data and machine learning, have given rise to new ‘digital’ solutions that can improve transparency and accountability in medicines procurement and supply chain management [18]. Many of these technologies have yet to be fully explored from an interdisciplinary perspective for their potential utility to improve access to medicines and combat pharmaceutical corruption. In this review, we examine the key characteristics and types of digital technology that can be used to improve transparency and accountability to combat corruption in medicines procurement. Based on these findings, we synthesize some best practices for medicines e-procurement systems, describe new and untested technologies on the horizon, and formulate a few key recommendations moving forward.

## Methods

**Figure 1.**
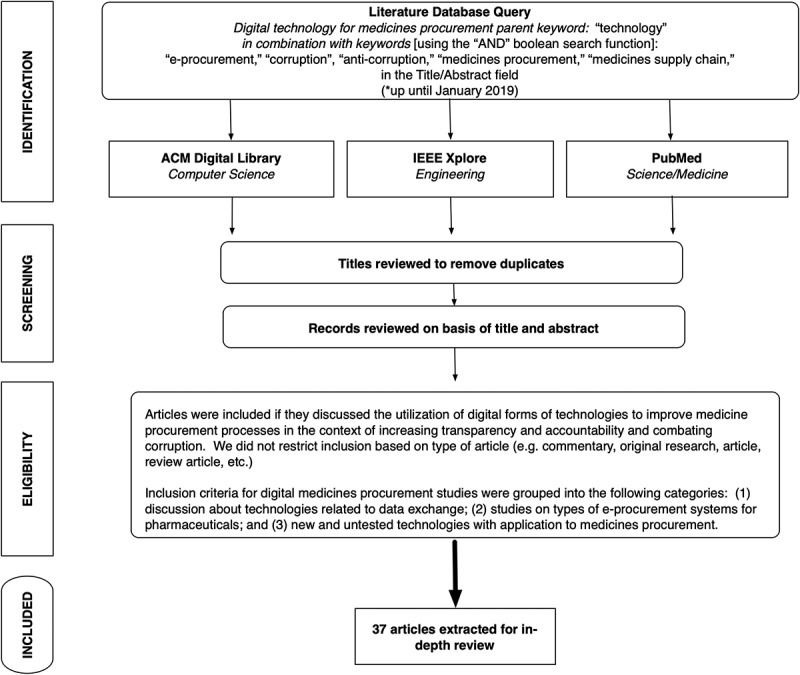
Summary of literature review methods.

In order to better understand the digital ecosystem for medicines procurement and its potential impact on corruption, we conducted an interdisciplinary literature review for journal articles, original research, conference papers, case reports, technology reviews, commentaries and news reports that were indexed in three scholarly databases (see summary of literature review methodology in Figure 1). This included conducting search term queries on PubMed (Medline), IEEE Xplore, and ACM Digital Library. We chose these databases based on the interdisciplinary nature of this assessment which requires a review of the science/health literature (from PubMed-indexed journals that cover life sciences and biomedical topics); studies on information, communication and engineering technologies (from IEEE Xplore-indexed articles that focus on scientific and technical content published by the Institute of Electrical and Electronics Engineers [IEEE]); and research on advances in computing sciences (from ACM Digital Library, which indexes various journals, conference proceedings, technical magazines, newsletters and books in the computing literature.)

We limited our searches to English-language articles. Our search queries constituted several keywords, including terms associated with procurement and digital technology combined with search terms associated with transparency and anti-corruption initiatives. Keywords were queried in the Title/Abstract field using advanced search function settings for PubMed, IEEE Xplore, and ACM Digital Library databases. Our definition of ‘digital technology’ focuses on concepts that primarily pertain to Internet-based communications, including online portals and management systems, supply chain tools, and electronic databases. We accepted articles from a wide range of time periods, as the nature of Internet-based solutions to medicines procurement imposes a self-limiting timeframe when such technologies became available. We generally placed a heavier emphasis on articles published in the last 10 years, though several older articles provided context for the evolution of digital technologies and were included in our review. Searches were conducted from October 2018 – January 2019.

After our initial search results, we applied inclusion and exclusion criteria that filtered results by reviewing the abstracts of extracted articles. We excluded articles whose discussion was unrelated to the use of digital technology for increasing transparency in the procurement of medicines. Additionally, when cited/referenced in a published study and relevant to the aims of this research, we retrieved and reviewed information from technical reports, reports from government agencies, information from non-governmental organizations/trade associations/solution providers/supply chain companies, and information from government and regulatory agency websites (grey literature). We examined these sources for existing (e.g. those already being deployed) and emerging technology (e.g. those still being experimented with or are currently under development) identified and/or referenced in our literature review. We included discussion from these sources for the purposes of describing key types of technology, characteristics of technology, and a few case studies to illustrate their use and application.

## Results

Overall, the academic literature in health, engineering, and computational sciences addresses the challenges of medicines procurement and use of technology in different ways based on the 37 articles retrieved in our literature review. Searches on IEEE Xplore and ACM Digital Library resulted in studies primarily focused on the technical specifications of existing and emerging technology as it relates to medicines procurement and more broadly the global medicines supply chain, but in many cases these articles lacked tangible application or translation of the technology’s impact on actual health outcomes or health systems. Conversely, the health literature retrieved in PubMed primarily focused on assessments or the implications of technologies on patients and population health outcomes, but often lacked detailed technical information and rarely included studies exploring proof-of-concept, experimental, or unvalidated technologies.

Overall, the vast majority of retrieved studies focused on the topic of e-procurement (described in detail below), with fewer studies devoted to other procurement digital solutions, and even fewer that discussed emerging technologies that were not directly incorporated into e-procurement systems. We describe each of these categories below by beginning with a discussion of how written forms (paper-based) of procurement have evolved into electronic data delivery systems and later into more complete e-procurement solutions (see Table 1 for summary). A full summary of extracted articles is also available in Table 2. Finally, we end with a discussion of new technologies for medicines procurement that are emerging.

**Table 1.** Summary of existing and emerging digital procurement technologies.

Technology Type and Status	Description	Use in Pharmaceutical Supply Chain and Procurement	Features	
			Data Exchange	Integration of other technology solutions
***Electronic Data Interchange (EDI)*** *[status: legacy technology]*	Method of transferring business transactional data on private networks using a standardized/ structured format. Still a widely used electronic message data format and largely replaced ‘paper-only’ based transactions.	Pharmaceutical trading partners engaged in procurement or in pharmaceutical supply chain management exchange order information. Generally data transfer between two parties.	Yes	Generally no
***e-Procurement Systems*** *[status: in process of being adopted technology]*	Use of electronic methods to conduct transactions between awarding authorities and suppliers, often using digital platforms and web-enabled systems.	e-procurement systems are used to consolidate the processes of purchasing, sourcing and tendering, and contracting and ordering of pharmaceutical procurement and can be used by multiple healthcare actors.	Yes	Yes
***Blockchain*** *[status: untested and emerging technology]*	Emerging distributed digital ledger technology that can help track transaction data of assets as secured through cryptography	Currently being explored as tool to enhance pharmaceutical supply chain management and could be used in conjunction with EDI or e-procurement systems. Potential to be used by multiple actors in consortia.	Yes (though transaction data may be off-chain)	Yes

**Table 2.** Summary of articles extracted for review.

Article Name/Authors	Methodology	Key Finding
**Andries 2007** E-procurement in the hospital industry: A feasibility study	Case series	Both demand- and supply-side issues decrease the likelihood of e-procurement hospital implementation.
**Azmi and Rahman 2015** E-procurement: A tool to mitigate public procurement fraud in Malaysia?	Interviews	Malaysian procurement officers indicated that e-procurement mitigates fraud in public procurement.
**Baghdad-Sabeta and Serhan 2010** Good Governance for Medicines programme: an innovative approach to prevent corruption in the pharmaceutical sector	Case series	A review of country-level implementations of the WHO’s GGM programme indicate that successful prevention of corruption is bolstered through practices that primarily institutionalize GGM in government structures/plans.
**Breen 2005** Improving the pharmaceutical supply chain	Questionnaire	UK pharmacy staff indicated that electronic data interchange integration can instigate quality improvements.
**Clauson et al 2018** Leveraging blockchain technology to enhance supply chain management in healthcare	Literature review	The literature suggests that implementation of blockchain technology may yield improvements to the security of the health supply chain.
**Cohen and Montoya 2001** Using technology to fight corruption in pharmaceutical purchasing: Lessons learned from the Chilean experience	Perspectives	Chile’s CENEBAST, utilizing centralized e-procurement with a clear incentive structure, has the potential to minimize pharmaceutical corruption.
**Cullen and Taylor 2009** Critical success factors for B2B e-commerce use within the UK NHS pharmaceutical supply chain	Questionnaire	UK pharmacy staff indicated five critical success factors for successful e-commerce use: system quality, information quality, management and use, assurance and empathy, and trust.
**DFID 2010** Addressing corruption in the health sector	Perspectives	E-procurement tools are concrete examples for how information technology can be used to increase accountability and transparency.
**European Commission 2013** Study on corruption in the healthcare sector	Interviews	Procurement corruption is widespread in EU member states, but no single policy is likely to work for all EU member states.
**Hakvoort 2004** ebXML and its impact on conventional business information systems	Technical write-up	Unlike EDI, ebXML is more likely to facilitate free exchange of information for business transactions.
**Hidalgo et al 2010** E-procurement in hospitals	Case study	The use of e-procurement in a psychiatric hospital in Spain yielded several financial and managerial improvements.
**Humphreys 2015** E-procurement in support of universal health coverage	Perspectives	E-procurement has wrought benefits in several countries around the world and will also be beneficial for Kenya.
**Hussman 2011** Addressing corruption in the health sector: Securing equitable access to health care for everyone	Perspectives	E-procurement can be used by governments to discourage corruption and improve efficiency.
**Ketikidis and Stalidis 2010** Applying e-procurement system in the healthcare: the EPOS paradigm	Questionnaires	EU healthcare respondents identified ten factors that can challenge e-procurement efficacy: time, cost, payment, training, security, publicity, interoperability, human factor, quality check, and EU market fragmentation.
**Kim and Shunk 2004** Matching indirect procurement process with different B2B e-procurement systems	Literature review	There are primarily four dominant B2B e-procurement system types: buyer-centric, supplier-centric, third-party, and end-to-end document exchange.
**King Jr et al 1994** System for ordering items using an electronic catalogue	Patent	Early IBM system that describes a computer system to search catalogues of product offerings and submit electronic purchase requisitions.
**Kohler et al 2015** Does pharmaceutical pricing transparency matter? Examining Brazil’s public procurement system	Financial analysis	Brazil’s Preços em Saúde initiative to increase drug pricing transparency did not decrease prices, potentially calling into question the effectiveness of e-procurement initiatives.
**Kulp et al 2006** Using organizational control mechanisms to enhance procurement efficiency: How Glaxosmithkline improved the effectiveness of e-procurement	Financial analysis	Analysis of GSK financial data finds that noncompliance is a major barrier to realizing savings from e-procurement use.
**Kuo et al 2017** Blockchain distributed ledger technologies for biomedical and health care applications	Literature review	Blockchain distributed ledger technology represents a potential benefit to the healthcare and biomedical sectors.
**Mackey et al 2016** The disease of corruption: views on how to fight corruption to advance 21st century health goals	Perspectives	Multiple members of a multidisciplinary team cite e-procurement as key tool in global health anticorruption.
**Mackey and Nayyar 2017** A review of existing and emerging digital technologies to combat the global trade in fake medicines	Literature review	Mobile technologies, RFID solutions, advanced computational algorithms, online pharmacy verification, and blockchain technology are digital technologies that can be successfully leveraged to combat counterfeiting.
**Mackey et al 2019** ‘Fit-for-purpose?’–challenges and opportunities for applications of blockchain technology in the future of healthcare	Perspectives	A multidisciplinary health technology team proposes that the design of blockchain technology must take into consideration the needs of its diverse stakeholders.
**Mettler and Rohner 2009** E-procurement in hospital pharmacies: An exploratory multi-case study from Switzerland	Case series	Analysis of Swiss hospitals indicates that lack of both technological and organizational change underlie slow adoption of e-procurement.
**Miroslav et al 2014** Semantic technologies on the mission: Preventing corruption in public procurement	Technical write-up	Automatic semantic technology may enable the Serbian w-procurement system to overcome a high burden of corruption.
**Moisil and Jitaru 2006** E-health progresses in Romania	Policy review	The Romanian e-procurement system can greatly benefit its healthcare system but requires investment in communication infrastructure.
**Schøll and Ubaydi 2017** Impact of technology on corruption	Financial analysis	The Ukranian e-procurement system has resulted in decreased prices and improved transparency.
**Seidman and Atun 2017** Do changes to supply chains and procurement processes yield cost savings and improve availability of pharmaceuticals, vaccines or health products? A systematic review of evidence from low-income and middle-income countries	Literature review	Centralized procurement systems have been found to decrease prices of health products in a number of subnational and national areas, including both high-income countries as well as low and middle-income countries.
**Sigulem and Zucchi 2009** E-procurement in the Brazilian healthcare system: The impact of joint drug purchases by a hospital network	Financial analysis	E-procurement resulted in notable savings for drug purchases in seven Brazilian university hospitals.
**Singer et al 2009** Does e-procurement save the state money?	Financial analysis	Chile’s e-procurement system resulted in both price reductions and administrative cost savings.
**Smith and Flanegin 2004** E-procurement and automatic identification: Enhancing supply chain management in the healthcare industry	Perspectives	Automatic Data Identification and Data Capture (AIDC) technology can bolster the benefits produced by e-procurement systems.
**Swatman and Swatman 1992** EDI system integration: A definition and literature survey	Literature review	The successful use of EDI systems must involve organizational, social, managerial, and strategic considerations on top of those related to communication.
**UNDP 2011** Fighting corruption in the health sector: Methods, tools, and good practices	Literature review	The Chilean system is a good example of how to effectively use e-procurement to combat corruption in procuring drugs.
**Vlasov and Parkhimovich 2014** Development of the open budget format	Technical write-up	The OCDS-based Open Budget Format can help Russian e-procurement initiatives to work with financial data.
**Waning and Vian 2008** Transparency and accountability in an electronic era: The case of pharmaceutical procurements	Perspectives	Identifying price outliers and establishing country benchmarks can help e-procurement bolster transparency.
**WHO 2009** Measuring transparency in the public pharmaceutical sector: Assessment instrument	Technical write-up	Questions relating to procurement methods, quantities, tenders, and management information systems are part of a survey instrument that can assess pharmaceutical sector transparency.
**WHO 2010** World Health Report: Chapter 4, more health for more money	Perspectives	Inefficiencies from the use of substandard/counterfeit medicines and the underuse of generics can stem from weak procurement systems.
**Wickramasinghe and Fadlalla 2005** A framework for assessing e-heatlh preparedness	Perspectives	Internet-based e-procurement is superior to other procurement systems due to decreased time and geographic barriers.

### Brief history of paper to electronic procurement

Procurement practices have existed for thousands of years (e.g. records of public procurement have existed for at least five thousand years since the time of the ancient Egyptians) and have more recently evolved in the past century to include modalities that extend beyond paper-based procurement processes to incorporate different forms of technology or electronic means for carrying out procurement practices [19]. Compared to forms of paper and written procurement, electronic forms of procurement have a much shorter history.

E-procurement, short for ‘electronic procurement,’ is the term used today to describe digital methods to procure items and commodities primarily using information or Internet-based technology platforms [20]. Its origins can be traced to the development of electronic communication transmitted over analog signals, starting with the popularization of the telegraph and the development of the telephone in the nineteenth century. As the procurement of goods through telephony required the transmission of electric signals, purchase orders via telegraph/telephone were among the earliest forms of static ‘e-procurement.’

Later, the transference of transactional information on private digital networks came to be known as an ‘Electronic Data Interchange’ (EDI) and was popularly used by customs authorities as a means of tracking and coordinating the flow of goods through ports [21]. By the 1980s, the predominant procurement modality shifted from telephone-based ordering relying on analog signals to EDI-based ordering relying upon digital signals [22]. In 1990, IBM filed a patent titled ‘System for ordering items using an electronic catalogue,’ in which customers could use a computer system to search catalogues of product offerings and submit an electronic purchase requisition that would be transmitted to the business and routed to the supplier [23]. This patent was arguably the precursor for what is now the predominant method of procurement: a shift from private digital networks (i.e. EDIs) to Internet-based networks and platforms. This adoption to what can be considered as modern forms of e-procurement, was accelerated by the establishment of the World Wide Web Consortium (W3C) in 1994, which proposed international standards for Internet communications.

Today forms of EDI data exchange remain popular and continue to be widely used globally among different industrial sectors, including for healthcare and medicines procurement. However, the emergence of the Internet as a global marketplace further enabled by web-based applications, high speed and large data transfer, and mobile and connected devices, has given rise to e-procurement systems that are more holistic as they can be versatile, adaptive, and can interface with other technologies.

### Medicines e-procurement systems

#### Key characteristics

We now shift our discussion to the current interdisciplinary literature on e-procurement systems for medicines. In this section, we analyze how the literature informs adoption, implementation, and evolving characteristics of e-procurement systems. Broadly speaking, e-procurement systems have additive components that go beyond EDI data exchange for order fulfillment including (1) ability to increase transparency of data to different actors; (2) enable greater automation of procurement processes and transactions; and (3) integration of other anti-fraud and anti-counterfeit technologies.

First, enhancing transparency of medicines procurement data and decision-making is an inherent characteristic of e-procurement systems and is also a driving factor in their potential to combat corruption and fraud in medicines procurement processes [24]. The fundamental advantage of e-procurement systems is their ability to digitize readable, writeable, storable, and searchable information for different procurement phases (e.g. pre-bidding, bidding, and award stages) and processes (e.g. issuing a request for proposals/tender, pricing of tenders, award decision-making). Accordingly, government officials have lauded e-procurement systems as avenues to increase transparency so as to decrease the potential for bid rigging [25].

Greater access to electronic procurement transaction data can also increase compliance to public procurement policies and laws by generating audit trails, and allow the public to scrutinize decisions and actions by enhancing availability and integrity of data under broader transparency and accountability activities such as e-government and open government initiatives [26]. Generating more open and accessible data in e-procurement systems can also provide critical data about bidding and award processes (including actual bidding processes and procedures, and results of successful bids) and drug acquisition prices, subsequently encouraging greater competition and deterring collusion and other forms of bid rigging.

Beyond transparency, studies reviewed also recognized the ability of e-procurement systems to incorporate new computational science methods to enable automation. Specifically, e-procurement systems have the ability to store electronic data fields with great detail on procurement transactions/decisions, which lends itself to the development of machine learning approaches that can detect fraud and abuse in tenders/bidding, similar to data mining techniques used for healthcare claims data [18]. On example of the application of machine learning in e-procurement found that artificial intelligence techniques could optimize pharmaceutical pricing and acquisition to find ‘best buys’ of pharmaceutical products [27]. However, this example enables cost savings, and our review did not uncover specific empirical studies of artificial intelligence within e-procurement systems used to detect corrupt practices.

E-procurement systems also have the ability to enable other forms of procurement, anti-counterfeiting and fraud detection technologies in a more complete and integrated solution. For example, articles noted that e-procurement strategies integrating automatic data identification and data capture (AIDC) technology and mobile devices could be used to effectively track pharmaceuticals throughout the supply chain and even for patient consumption of pharmaceutical products [28,29]. AIDC consists of a host of different technologies (e.g. bar codes, magnetic stripes, radio-frequency identification [RFID], smart cards) operating together in an automated fashion to identify objects, collect data from them, and then input them into computer systems, all elements that lend well to supply chain track and trace solutions. Today, AIDC and mobile technologies have experienced mixed levels of adoption and implementation in the medicines supply chain, with the widespread use of barcodes, but lower utilization of more advanced technologies such as RFID largely due to the higher costs of these technologies [18]. In contrast, EDI systems that simply transfer transaction data cannot traditionally incorporate additional data from anti-counterfeiting or security technologies, though represents a more established and standardized technology that is easier to adopt.

#### Importance of cost-savings generation

Though key characteristics of e-procurement systems demonstrate how these systems can be used to increase transparency, enable automation, and integrate other forms of technology, arguably the most important function of an e-procurement system is its ability to generate cost-savings [29]. In fact, an e-procurement system’s ability to reduce procurement costs is likely the single most important factor for medicines e-procurement systems adoption [28].

For example, a 2006 review of e-Health interventions in Romania found that e-procurement was used by the Ministry of Health for the acquisition of medicines for HIV/AIDS, tuberculosis, oncology, nephrology, diabetes, hematology, and psychiatry [30–34]. One of the key factors for this technology adoption was the expected cost savings of some US$1.5 million [30]. Cost-savings can come in the form of process improvements, such as a 2010 study describing a pilot e-procurement system in Greek, Spanish, and Belgian hospitals [35]. This system reportedly halved the time required to create a list of goods and reduced the time to prepare tenders from 1 h to 15 min, again emphasizing the focus of cost-saving objectives for initial e-procurement projects [35]. A case study in Spain also reported cost savings of 20%, totaling €121,063.12 for the period studied, for the purchase of healthcare products from a Catholic psychiatric hospital in Barcelona [36]. Further detailed examples of the tie between medicines e-procurement systems and cost-saving benefits are provided in case studies of Chile and Brazil (Text Box 1 and 2).

**Table:** 

**Text Box 1 (Chile Case Study**): The *Central Nacional de Abastecimiento* (CENABAST) is the centralized Chilean medicines e-procurement agent and distributor. In the early 2000s, CENABAST was reformed from a largely non-transparent system characterized by stock-outs and inefficiencies to a transparent e-procurement system requiring use of the website for Chile’s Public Sector Contracting and Procurement Division, www.chilecompra.cl, known as ChileCompra (also used for non-medicines public procurement in Chile). When Chilean health facilities need to procure medicines, they post tenders onto ChileCompra. By requiring public health facilities to participate in ChileCompra, CENABAST standardizes medical procurement and facilitates data transparency. Savings from use of the reformed system were estimated to be between 5 to 7% [58]. In addition, a later analysis of ChileCompra (not exclusive to medicines), also found analogous benefits from the use of a transparent e-procurement system [59]. Specifically, the 2007 purchases of 885 Chilean State agencies were assessed. For users of this system, the authors found overall price reductions of 2.65% and administrative cost savings of approximately 0.3% across purchases of US$4.5 billion [59]. This case study demonstrates that though transparency in e-procurement is key to anti-corruption efforts, the benefits of such systems are largely measured in cost savings, either from process improvements or lowering commodity prices, and not, for example, anti-fraud (e.g. the number of illegal bids or tenders detected) or public health (e.g. stock outs) measures.

**Table:** 

**Text Box 2 (Brazil Case Study**): A 2009 study of an e-procurement system introduced to nine Brazilian hospitals revealed a decrease in the price of acquiring medicines of over ten percent for 47% of medicines assessed [60]. The authors noted that the e-procurement platform was chosen “to ensure transparency for the mediation of commercial transactions—benefiting purchasing hospitals on the one hand with the possibility of expenditure rationalization, and suppliers on the other and with sales on a larger scale” [60]. It should be noted that the e-procurement system was intended to provide a way for these seven hospitals to jointly purchase medications, similar to a group purchasing or volume/pooler purchasing arrangement. Hence, price reductions may be attributable to greater purchasing leverage, in addition to improved transparency. However, inconclusive findings on cost-savings were reported in a 2015 analysis of *Preços em Saúde* (BPS), Brazil’s drug procurement system, which assessed changes in the prices of drugs whose acquisition prices were made publicly available on the Internet [61]. The study assessed purchases made by all federally funded hospitals in two socioeconomically distinct Brazilian states, Pariba and São Paulo, over a five-year period. Importantly, purchases analyzed in this study did not have to be made using an e-procurement system. The analysis found that an Internet-based strategy to improve pricing transparency did not lead to statistically significant reductions in actual purchase price, suggesting that merely publishing purchase prices for medications may be insufficient to reduce prices. These findings may indicate that transparency alone is insufficient for cost-saving purposes, and that implementation of e-procurement systems that require full bidding and fulfillment via electronic means are key enabling factors.

Yet, when taking into consideration the cost of technology adoption, training, ongoing maintenance, integration and implementation, and information security, estimates such as those in Romania and other countries may be overly optimistic in projecting actual cost savings [30–34]. For example, a 2006 study on implementation of e-sourcing tools used internally by the global pharmaceutical company GlaxoSmithKline (GSK) found that the main challenge in realizing cost savings from these systems was appropriately identifying and addressing noncompliant purchases (when there is a failure to comply with the terms and conditions of procurement contracts) [37]. Specifically, GSK estimated that they would have realized ‘between $80 and $120 million’ in extra savings if noncompliance in procurement were eliminated. In computing this estimate, GSK analyzed a sample of their expenses to determine compliance, quantified lost savings for noncompliant procurements, and determined reasons for noncompliance. They found that ‘lack of information’ of compliant procedures was the top reason for noncompliant procurement. To remedy this, GSK created an internal online portal to allow company employees to find authorized vendors offering items at contracted rates. Follow-up data showed a decrease in noncompliance after this intervention. Therefore, the creation of a searchable, user-friendly means of communicating compliance information was a critical adjunct in enabling savings to be realized from existing e-procurement mechanisms.

Though e-procurement systems that can facilitate enhancing transparency, automation, technology integration, and enable cost reduction are being explored and tested, more elusive goals of directly detecting, reporting, and enabling enforcement against fraud and corruption in medicines procurement appear to be less studied. Overall, there are few examples of e-procurement systems that successfully address *both* improving cost-savings and reducing corruption, though the case study of Ukraine is a notable exception (see **Text** Box 3). In this sense, adoption of e-procurement systems is largely driven by cost-savings estimates but has failed to directly link anti-corruption measurements to also improving cost-savings either through preventing or detecting fraud, or improving overall health outcomes. Lack of this activity may indicate that non-technology barriers need to be taken into consideration and could be preventing more complete adoption of e-procurement systems as an anti-corruption tool.

**Table:** 

**Text Box 3 (Ukraine Case Study**): One of the most publicized examples of e-procurement success for pharmaceuticals is Ukraine’s public procurement system ProZorro. ProZorro was developed in 2015, in collaboration with Transparency International, specifically as a response to a public procurement system acknowledged in the Ukraine to suffer from widespread corruption [62]. Its key features include a fully online system following the Open Contracting Data Standards (OCDS) guidelines developed by the World Bank. While not exclusive to medicines, procurement on ProZorro includes government e-procurement of medicines. An economic assessment of ProZorro found that it had a positive impact on exposing corruption and decreasing prices for the Ukrainian government [63]. This study found that decreases in prices were greater for simpler services (i.e. transactions for homogenous goods like natural gas) than for more complex services (e.g. dam engineering or specialized research). The authors argue that the difficulty of detecting collusion increases with the complexity of the service being procured, which explains why ProZorro’s impact on reducing prices could be diminished for transactions for complex goods. Different from previous case studies, ProZorro provides early support for the dual role of medicines e-procurement systems in *both* improving cost-savings and reducing corruption by creating more fair and competitive processes through a centralized system.

### Non-technology considerations and barriers for e-procurements systems

Though the technology behind medicines e-procurement systems have the ability to facilitate enhanced transparency, accountability, and anti-corruption approaches, system design and factors outside of technology considerations also need to be addressed. For example, a 2009 study of business-to-business (B2B) e-procurement systems used by the UK’s National Health Service (NHS) pharmaceutical supply chain found that *both* system design (e.g. centralization of e-procurement systems) and the need to establish trust and confidence among users were critical success factors in e-procurement system implementation [38].

Related to system design, the benefits of centralizing e-procurement systems have been studied in the context of improving logistic management processes and enabling cost-savings [39]. For example, a 2016 systematic review assessing 38 articles found evidence that centralized digital *and* non-digital procurement solutions result in direct cost savings, reduced stock outs of pharmaceuticals, and increased drug availability for the public [40]. This study also found that Vendor Managed Inventories (i.e. logistic systems where vendors can fill orders based on readily available inventory information) can also save costs in conjunction with broader adoption of e-procurement.

Further, though there is evidence that medicines e-procurement solutions exhibit many advantages over traditional forms of electronic data transfer (i.e. EDI), overall adoption of e-procurement systems appears to be slow, even in highly developed countries [41]. For example, a 2009 analysis of three case studies of hospitals in Switzerland found slow adoption of e-procurement and advocated for specific technologies that can interface with e-procurement systems, including RFID, computerized physician order entry (CPOE), unit dose system of medication distribution (UDDS), and supplier relationship management (SRM) [42]. The integration of these additional technologies/solutions may encourage broader e-procurement adoption by increasing the utility of systems that are focused on enabling cost-saving approaches.

Another challenge in medicines e-procurement adoption has been the resilience of legacy procurement modalities and user perceptions about the limitations of what e-procurement systems can do. A 2004 synthesis of interviews with materials management officials of Oregon hospitals found that the perceived inability of management to negotiate lower prices with suppliers was a major barrier to the adoption of e-procurement [43]. Conversely, a 2004 study analyzing case studies of the hospital–supplier relationship found that adoption of e-procurement resulted in hospitals’ consolidation of suppliers and a consequent deepening of hospital–supplier relationships [44]. This study indicates that e-procurement can enable hospitals to better assess the relative quality of suppliers, and also that e-procurement can facilitate long-term collaborations between hospitals and suppliers which may lead to improved quality of care. While dated, these studies provide insight into potential challenges in other countries that are beginning their cycles of medicines e-procurement system exploration, adoption, implementation, and management, particularly in settings where the introduction of widespread and reliable information and communication (ICT) infrastructure is still developing.

Finally, technical standards must change as organizations transition from EDI to e-procurement systems. Electronic business transactions, including those for e-procurement, once relied upon technical standards relating to EDI, specifically the United Nations/Electronic Data Interchange for Administration, Commerce and Transport (UN/EDIFACT), approved by the International Organization for Standardization (ISO) as ISO 9735 [45]. With the Internet facilitating the transition of electronic communication from private networks to open networks, the business community migrated largely toward technical standards relating to XML (a markup language for encoding documents largely used for Internet sites and applications).

Hence, technical standards setting will be critical to e-procurement adoption as it will increase interoperability between different systems, encouraging greater data exchange, and ideally enhancing transparency that can drive future anti-corruption solutions with robust data sharing. In 2015, the World Bank issued the Open Contracting Data Standard (OCDS), a guideline for government procurement, which requires public accessibility of procurement information from planning to the end of a contract with data and processes ranging from tenders to also including bids, participants, and decision-making processes [46]. The OCDS marks a critical step in e-procurement standards setting and has the potential to pave the way for greater adoption, including in the case of medicines public procurement. However, challenges regarding implementation of this highly technical standard (particularly in low technical capacity settings), adoption from both public and private sectors, ensuring consistent and quality data, and enabling users to actually use data to improve e-procurement and root out corruption are all critical factors not fully addressed [11].

### Blockchain: an emerging digital procurement technology?

In addition to well-established forms of EDI and e-procurement systems used in medicines procurement, a few emerging technology solutions are gaining attention for their potential to fight corruption including the use of mobile devices, use of machine learning for supply chain optimization and pharmaceutical diversion, and blockchain technology [18]. Importantly, these technologies would operate in conjunction with (not separate from) e-procurement systems and open government initiatives, therefore integrating with or augmenting existing e-procurement approaches. One such technology that is receiving a great deal of attention as a transparency, trust, and data provenance tool is blockchain which includes distributed ledger technology.

Blockchain is a relatively new form of technology that is most widely associated with the bitcoin cryptocurrency that popularized its use. In general, blockchain technology is shared, distributed digital ledger technology (copies of electronic ledgers of transaction data that are hosted on multiple computers/nodes) that can help to track the recording of data, transactions, and assets (including medicines, healthcare payments, or global health aid funding) [47]. Importantly, blockchain technology is also secured by cryptography (advanced encryption) to ensure data immutability, provenance, and enhance integrity and trustworthiness of such data [48,49]. Simply put, a blockchain allows the formation of ‘blocks’ of related and time-stamped transaction data from multiple trusted parties connected by a hash chain (which links blocks of data from transactions to each other), which when taken as a whole, can give you a complete picture of the entirety of the transaction or, for example, the procurement or supply chain records associated with pharmaceuticals. Currently, there are several use cases for blockchain being explored in healthcare, including clinical trial management, genomics, electronic health records, consumer health applications, and most notably, supply chain management [47].

In the context of medicines procurement, a private or ‘business’ blockchain could be used to better validate transactions across the supply chain by ensuring that only ‘trusted’ partners participate and can validate information related to procurement, distribution, and dispensing of a medicine. If fraudulent medicines supply transaction information is introduced, participants can identify it and then reach consensus that it is not valid (as a distributed ledger can be viewed simultaneously by all parties that have the permissions to view it) [18]. Inherently, when designed properly, blockchain technology can enable transparency and trust in supply chain data (i.e. the ledger is shared, data security and provenance are supported by cryptography, and the parties agree on how to reach consensus on validity of transitions), all principles of good governance in medicines which can help fight corruption [50].

Extending this to medicines procurement, a blockchain-enabled e-procurement system could have a permissions structure that makes certain data available to all participants on a blockchain (increasing transparency) while also validating who can participate, could create an immutable and tamper-evident log of the history of supply chain transactions for enhancing audit or procurement decisions for compliance purposes, and could create more efficient supply chain processes through the use of smart contracts while also interacting with other anti-corruption technologies (such as AIDC). However, blockchain design, adoption, and implementation are still in early stages, with lingering practical and technical considerations needing to be explored though some researchers are currently prototyping use cases to fight falsified medicines [51]. Similar challenges of governing data sharing and establishing internationally agreed standards for blockchain that align with medicines e-procurement systems and OCDS will be critical for future adoption, along with making sure these solutions are compatible and bring added-value for pharmaceutical supply chain track and trace policies such as the U.S. Drug Supply Chain Security Act and the EU Falsified Medicines Directive [49].

## Limitations

This review has certain limitations. First, our study relies primarily on published literature to assess the current characteristics, levels of adoption, and strengths and limitations of digital technologies associated with medicines procurement and applications to anti-corruption. However, our literature review detected a relatively small number of articles and though the grey literature supplemented these results, our primary results nevertheless relied upon scientifically based sources of information in journals. This may limit the scope and generalizability of the study, particularly since technology adoption and implementation may not be reported in the literature or such information could be construed as confidential. Second, some of the sources reviewed were published prior to 2010 and may no longer be current to more recent technology characteristics and features. These limitations point to the acute need for more recent research assessing the actual impact of identified technology on both improving pharmaceutical procurement processes and its ability to enhance transparency, accountability, and acting as a critical tool for anti-corruption. Such research would serve as an important evidence-base for adoption and best practices for anti-corruption technology implementation.

## Discussion

This study conducted an interdisciplinary assessment of different digital tools that are being used to improve transparency and accountability to fight corruption in medicines procurement. Though our search was not limited to specific types of technology, the vast majority of the literature focused on medicines e-procurement systems. This is not particularly surprising given that e-procurement systems intersect with key characteristics associated with good governance for medicines as discussed by Kohler and colleagues (e.g. promoting transparency and accountability), broader open government and e-government initiatives (e.g. ensuring greater citizen participation and public oversight), and activities aimed at deterring corrupt activities as discussed by Kohler and colleagues in this special issue.

However, in order to be successful, research indicates that the ability for e-procurement systems to generate cost-savings is a key driver of adoption and utilization and that anti-corruption objectives may be secondary. In this sense, anti-corruption advocates should be explicit in tying cost-saving measurements as a measurable output of good governance and anti-corruption approaches when using digital technologies such as e-procurement systems in order to better encouraged shared goals of adoption. This should be supported by purposeful measurement of the effectiveness of data points collected in an e-procurement system that can simultaneously measure cost savings and potentially act as a proxy indicator or risk signal for corruption and fraud and abuse.

This could be accomplished by adopting a set of well-defined anti-corruption indicators specific for pharmaceutical procurement into Health Technology Assessments (HTAs) also within the context of broader support for Universal Health Coverage (UHC) as has been used in other sectors of public health [24,52,53]. HTAs can also be informed by existing good governance and anti-corruption initiatives, such as the WHO’s Good Governance for Medicines tool that could be adopted specifically for anti-corruption technology assessments [16,54,55]. By taking these steps, stakeholders can better illustrate why increasing transparency, accountability and fighting corruption in medicines procurement are primary outcomes and measurable objectives in how e-procurement systems lower healthcare and public expenditure costs, while also enhancing access to medicines, and improving patient safety.

Key design elements for e-procurement systems are also important in future considerations. As a general rule, e-procurement systems should be designed with a focus on establishing trust and confidence among users and related stakeholders that go beyond the technological functions alone. Further, shared benefits that can be generated by centralized procurement systems can result in further cost benefits (through pooled procurement), enable better compliance (if governed by a central authority) and encourage data interoperability. In order to enable centralized systems, the importance of standards setting both domestically and internationally cannot be overstated.

Fortunately, the OCDS forms the basis for greater specialization and standard setting for medicines e-procurement systems, which can potentially be harmonized across different health system actors, public and private sector entities, and across different jurisdictions. Importantly, tools for facilitating the adoption of e-procurement systems from the World Bank and the Open Contracting Partnership are available [56,57]. All of these efforts will also need the support of increased and sustained investment for e-procurement systems adoption coupled with improvements to ICT infrastructure and coverage, particularly in low-to-middle income countries where pharmaceutical governance and/or rule of law may be weak [16].

Additionally, new and exciting technologies, such as machine learning and blockchain technologies, are on the horizon that can improve medicines procurement and concomitantly address corruption. Though our study discussed the promise of machine learning approaches to detect, characterize and report corrupt practices within the context of e-procurement, the actual deployment of artificial intelligence in medicines e-procurement systems appears to lack adequate representation and validation in the literature. Solutions that leverage blockchain technology to combat corruption, fraud and abuse in the pharmaceutical supply chain are also still in their infancy and unproven. Critical to more widespread adoption of new technologies will be purposeful peer-reviewed research that objectively validates the utility of such technology.

Importantly, these innovations cannot be successful without increased transparency and access to data that can be enabled by e-procurement systems and open government initiatives. In this sense, fundamental principles of good governance will serve to fuel the next generation of anti-corruption technology informed by more open data that can improve transparency and decision-making.

## Conclusion

Though evidence suggests that digital technologies, such as e-procurement, can be used as a part of broader strategies to improve transparency, lower costs, and potentially help deter corruption-related activities in the procurement of medicines, further research and evidence is needed to establish best practices and assessment of more case studies is needed to identify examples of real-world success and failure that can improve future implementation. Strong empirical evidence supporting further public and private investment in these systems to tackle the related health financing, patient safety, and public health consequences of corruption in medicines procurement remain underdeveloped, particularly in the context of how effective medicines e-procurement systems can be in preventing corruption and increasing access and affordability to essential medicines to further advance UHC. Despite these challenges, digital technology is here to stay and has the potential to modernize pharmaceutical procurement in a way that can also serve shared goals of saving money, lives, and improving health if deployed effectively.

## Paper context

A comprehensive review of e-procurement’s effect on reducing corruption had not been conducted. Our review indicates that corruption in medical procurement is widespread; there exists evidence from several disparate case studies of how e-procurement is likely reducing corruption, especially through available financial and transparency data; non-technological barriers exist to widespread e-procurement implementation; there are several distinct typologies of e-procurement systems; and emerging technologies like blockchain and machine learning show promise in helping to reduce corruption.
